# Copper Status in Alzheimer's Disease and Other Neurodegenerative Disorders: Genetics, Mechanisms, Neurophysiology, and Therapies

**DOI:** 10.4061/2011/903940

**Published:** 2011-12-15

**Authors:** Rosanna Squitti, D. Larry Sparks, Tjaard U. Hoogenraad, George J. Brewer

**Affiliations:** ^1^Department of Neurology, “Campus Biomedico” University, 00128 Rome, Italy; ^2^Department of Neuroscience, AFaR-Fatebenefratelli Hospital, 00186 Rome, Italy; ^3^Roberts Laboratory for Neurodegenerative Disease Research, Banner Sun Health Research Institute, Sun City, AZ 85351, USA; ^4^Department of Neurology, University Medical Centre Utrecht, 3584 CX Utrecht, The Netherlands; ^5^Department of Human Genetics, University of Michigan, Ann Arbor MI 48109, USA; ^6^Department of Internal Medicine, University of Michigan, Ann Arbor MI 48109, USA; ^7^Adeona Pharmaceuticals, Ann Arbor, MI 48103, USA

The present special issue is an attempt to explore some of the diverse aspects of copper involvement in the physiology of the brain. The contributions examine very hot topics, such as the relationship between copper and synaptic transmission or copper involvement in Alzheimer's disease (AD) and even include a computational model of copper binding to the amyloid precursor protein (S. Azini and R. Rouk 2011). In this model, the authors applied theoretical methods of computational chemistry to determine structure, binding affinities, and reduction potentials of Cu(II) and Cu(I) bound to models of the Asp1, Ala2, His6, and His13His14 regions of the amyloid beta peptide.

A bulk of new studies has been recently published on the role of copper in the biological processes subtending synaptic transmission, and three contributions on this subject are included in the special issue. Specifically, one contribution describes a study in a cellular model of oocytes, which investigated the connection between copper and the prion protein in neurotransmission mediated by ATP-evoked currents (R. A. Lorca et al., 2011). A study on mouse hippocampal slices investigates copper inhibition of glutamatergic NMDA receptor-independent long-term potentiation (N. L. Salazar-Weber and J. P. Smith 2011), and a human model explores the effects of systemic copper on glutamatergic mediated excitability in the primary somatosensory cortex (F. Tecchio et al., 2011). 

Since the original papers that have been fuelling a heated debate in the literature about the pros and cons of copper on cognition are rather scattered and discordant, the aim of the editors was to select contributions that all together would provide the reader with an adequate overview of the issue but would still leave the reader free to form personal and hopefully critical opinions. In this section, some of the papers give a good overview of the nonunivocal *in vitro*, animal and human findings which demonstrate the involvement of copper in AD (D. Kaden et al. 2011). Also, in their paper, Y. Manso and colleagues (Y. Manso et al., 2011) review the preclinical and clinical literature dedicated to therapeutic interventions aimed at delaying or possibly reverting AD progression via the modulation of brain copper levels (Y. Manso et al., 2011). In the same section, some reports are preliminary but still original in their content, as, for example, the paper dealing with the influence of water quality on the cholesterol-fed rabbit model, which suggests that the origin of the brain neurofibrillary tangles is possibly systemic and somehow related to disturbances to the blood brain barrier mediated by copper ingested through the diet (D. L. Sparks et al., 2011), as well as the study by S. Bucossi and coworkers (S. Bucossi et al., 2011) which demonstrates an association between single nucleotide polymorphisms of the Wilson's disease gene and AD. T. U. Hoogenraad discusses further the link between these two neurological disorders (T. U. Hoogenraad 2011), which share the presence of abnormalities in toxic nonceruloplasmin (also named “free”) copper, proposing Zinc therapy as a valid tool against AD, as it has already proven to be in Wilson's disease. The perspective that comes out of his contribution (T. U. Hoogenraad 2011), for some maybe visionary or prophetic, is probably somehow adherent to the author's attitude, pragmatic, as can be expected from a physician who has dedicated his career to Wilson's disease clinical care, but also filled with enthusiasm, as demonstrated by his attitude to be prone to ideas of shifting paradigms: he was one of the pioneer of Zinc therapy in Wilson's disease. 

With a totally different approach, G. J. Brewer (G. J. Brewer 2011) discusses AD from an epidemiologic point of view, advancing the hypothesis that ingestion of inorganic copper from copper-made plumbing or via consumption of copper-rich dietary supplements may have a role in AD generation. Finally, the issue presents also a study connecting higher levels of copper to gender differences and cognitive status (J. F. Quinn et al., 2011).

In conclusion, is copper good or bad in general for human health and in particular for cognition? Of course, the question is far from being answered in this special issue, but it is well discussed. These editors recommend reading carefully the diverse interpretations presented but also going back and read the original works which are at the basis of them. In fact, even though reliable, review articles often decrease the critical attitude of the reader, as recent publications about citation bias have pointed out [1].

*Rosanna Squitti* *Rosanna Squitti* *D. Larry Sparks* *D. Larry Sparks* *Tjaard U. Hoogenraad* *Tjaard U. Hoogenraad* *George J. Brewer* *George J. Brewer*

## References

[1] Schrag M, Mueller C, Oyoyo U, Smith MA, Kirsch WM (2011). Iron, zinc and copper in the Alzheimer's disease brain: a quantitative meta-analysis. Some insight on the influence of citation bias on scientific opinion. *Progress in Neurobiology*.

